# Efficacy of tofacitinib in the treatment of refractory palmoplantar psoriasis (non-pustular): case report and literature review^؆^

**DOI:** 10.1016/j.abd.2026.501357

**Published:** 2026-05-11

**Authors:** Paula Hitomi Sakiyama, Luciane Donida Bartoli Miot, Hélio Amante Miot

**Affiliations:** a Service of Dermatology, Hospital Universitário do Oeste do Paraná, Universidade Estadual do Oeste do Paraná, Cascavel, PR, Brazil; b Department of Infectology, Dermatology, Diagnostic Imaging and Radiotherapy, Faculty of Medicine, Universidade Estadual Paulista, Botucatu, SP, Brazil

Dear Editor,

Palmoplantar psoriasis is a chronic condition of the palms and/or soles, with a prevalence of 2%–40% among patients with psoriasis; however, specific epidemiological data are scarce. Although it can manifest in a localized form, it has a greater impact on quality of life compared to other areas. Its morphology varies from pustular lesions to erythematous-desquamative and hyperkeratotic plaques, or with overlapping lesions, being classified based on these phenotypes.^1^

Non-pustular palmoplantar psoriasis (NPPP), or hyperkeratotic psoriasis, represents one of the most challenging psoriasis phenotypes in clinical practice.^1^ It is a recalcitrant type, even with available therapeutic advances. Furthermore, the lack of parallelism in the clinical response of psoriasis vulgaris (PV) in relation to NPPP suggests a distinct pathogenesis, with reports of success with JAK inhibitors (JAKi) in the treatment of refractory NPPP.

In this text, the authors report a case of NPPP refractory to conventional and biological therapies with a complete response to oral tofacitinib. The authors also review analogous published cases and discuss relevant pathophysiological aspects.

A 48-year-old woman was diagnosed with NPPP (hands and feet), in addition to discrete plaques on the scalp, for 14 years. She reported previous treatments with topical corticosteroids, methotrexate (eight months), acitretin (eight months), adalimumab (five years), and secukinumab (three years), without a satisfactory response. On examination, she showed hyperkeratotic plaques and fissures on both soles (Fig. 1A). She reported being unable to perform physical activities or light walks.

**Figure 1:**
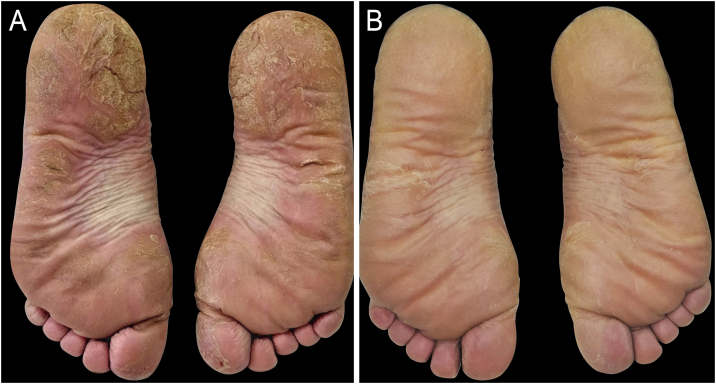
Non-pustular palmoplantar psoriasis treated with oral tofacitinib. (A) Pre-treatment: Physician's Global Assessment of Hands and/or Feet (hf-PGA) 4; modified Palmoplantar Psoriasis Area and Severity Index (m-PPPASI) 30; Palmoplantar Quality-of-Life Instrument (PPQLI) 38; and Dermatology Life Quality Index (DLQI) 7. (B) After 120 days of treatment: hf-PGA 2; m-PPPASI 3; PPQLI 23; and DLQI 0.

Histopathological examination of the palmar and plantar regions revealed chronic psoriasiform dermatitis, characterized by acanthosis with hypogranulosis and neutrophils in the epidermis, elongation of the interpapillary ridges with focal fusion, slight spongiosis, compact hyperparakeratosis with neutrophils, elongated capillaries, and lymphocytic infiltration in the papillary dermis (Fig. 2). The search for fungi by periodic acid-Schiff staining was negative.

**Figure 2:**
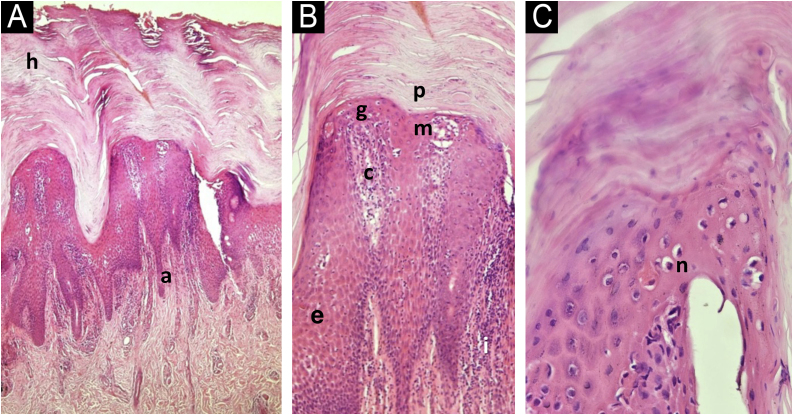
Histopathological examination of the plantar region. Panel A: Acanthosis, hyperkeratosis (h), elongation (a) of the epithelial ridges (Hematoxylin & eosin, ×100). Panel B: Proliferation of papillary capillaries (c), inflammatory infiltrate (i), parakeratosis (p), hypogranulosis (g), intraepidermal microabscess (m), discrete spongiosis (e) (Hematoxylin & eosin, ×200). Panel C: Hypogranulosis (g), with the presence of intraepidermal and subcorneal neutrophils (n) (Hematoxylin & eosin, ×400).

Considering the refractoriness and quality of life impairment, tofacitinib 5 mg orally, twice a day, was prescribed after clinical screening and complementary examinations. Clobetasol 0.05% ointment was maintained in the first month of treatment.

After 30 days, improvement in erythema, desquamation, and pain in the feet was observed, with complete remission of the hand lesions. The plantar fissures healed in 45 days, allowing a full return to physical activities.

After 60 days of treatment, she developed an upper respiratory tract infection, prompting temporary drug suspension for seven days. Upon reintroduction, she reported a mild headache, which subsided with a dose reduction to 7.5 mg/day, taken once daily.

After 120 days of treatment, she showed significant improvement in plantar lesions, full functionality, with resolution of difficulty and pain when walking, running, climbing stairs, and standing barefoot, as well as a reduction in severity scores (Fig. 1B). The patient has been followed for 12 months, with a current dose reduction to 5 mg/day. No changes were observed in laboratory tests during follow-up.

Topical medications and phototherapy constitute the first-line treatment in NPPP. However, most patients require systemic treatment, such as acitretin, methotrexate, and biologics. Biologics act on cytokines specific to the pathophysiology of psoriasis, including anti-TNFα, anti-IL-12/23, anti-IL-23, and anti-IL-17.^2^ The efficacy of these agents in NPPP, however, tends to be lower than in other forms of psoriasis.

Psoriasis is an immunologically based disease, with distinct inflammatory circuits among its phenotypes. Th1/IFN-γ inflammation predominates in NPPP compared to PV and palmoplantar pustular psoriasis (PPP), which shows inflammatory activity more associated with neutrophils.^3^

Tofacitinib is a JAKi, especially of JAK 1 and 3, and to a lesser extent JAK 2, which suppresses Th1, Th17 pathways, and innate immune cell signaling, reducing the expression of cytokines such as IFN-γ, IL-17A/F, IL-22, IL-23, and IL-36.^4^ This broad action may justify its effectiveness in refractory cases of NPPP.

Table 1 Reviews several reports of JAKi for the treatment of NPPP.^5^, ^6^, ^7^, ^8^, ^9^ There are also descriptions of therapeutic success in PPP, considered part of the psoriasis spectrum.^7^, ^10^

**Table 1:** Cases of palmoplantar psoriasis treated with oral Janus kinase inhibitors.

Report	Diagnosis	Study design	Sex/age	Previous treatment failure	Treatment	Response	Adverse events	Follow-up time
Valor-Méndez et al., 2021^5^	NPPP (palmoplantar), PV and PsA	Case report	Male, 55 years	Methotrexate, infliximab, secukinumab, and ustekinumab	Tofacitinib 5 mg 2× day	Improvement in 5 days; resolution in 6 months.	NR	6 months
Muzumdar et al., 2021^6^	NPPP (palmar) and PV	Case series (1/7)	Female, 65 years	Topical drugs, adalimumab, ixekizumab, and ustekinumab.	Tofacitinib XR 11 mg 1×/day	Resolution in 4 months	None	4 months
	NPPP (palmoplantar) and PsA	Case series (2/7)	Female, 63 years	Topical drugs, methotrexate, adalimumab, ixekizumab, secukinumab, and ustekinumab.	Tofacitinib XR 11 mg 1×/day	Improvement within 1 month; palmar resolution within 8 months and slight desquamation on the dorsum of the feet.	None	8 months
	NPPP (palmoplantar)	Case series (3/7)	Female, 57 years	Topical drugs, cyclosporine, methotrexate, adalimumab, certolizumab, guselkumab, ixekizumab, risankizumab, and secukinumab.	Tofacitinib XR 11 mg 1×/day	Almost complete resolution within 1 month.	None	1 month
	Mixed PP (palmoplantar) and PV	Case series (4/7)	Female, 45 years	Topical drugs, methotrexate, adalimumab, certolizumab, etanercept, guselkumab, ixekizumab, secukinumab, and ustekinumab.	Tofacitinib XR 11 mg 1×/day	Improvement within 1 month; resolution within 3 months.	None	3 months
	NPPP (plantar)	Case series (5/7)	Female, 69 years	Topical drugs, apremilast and methotrexate	Tofacitinib 5 mg 2×/day	Improvement within 3 months; palmar resolution and mild plantar hyperkeratosis within 6 months.	None	6 months
	NPPP (plantar) PsA	Case series (6/7)	Female, 61 years	Topical drugs, apremilast, methotrexate, guselkumab, and ixekizumab	Tofacitinib 5 mg 2×/day	Improvement within 2 months with slight residual plantar desquamation.	None	2 months
	NPPP (palmar) and PsA	Case series (7/7)	Female, 66 years	Topical drugs, methotrexate, ixekizumab, and secukinumab	Tofacitinib XR 11 mg 1×/day	Almost complete resolution within 1 month.	None	1 month
De Luca et al., 2024^7^	PPP (palmar) and PV	Case series (1/5)	Male, 33 years	Apremilast and secukinumab	Deucravacitinib 6 mg 1×/day and etanercept 50 mg/week	Exacerbation in weeks 4 and 16	NR	4 months
	PPP (plantar) and PV	Case series (2/5)	Female, 49 years	Phototherapy, fumaric acid, apremilast and cyclosporine	Deucravacitinib 6 mg 1×/day	Improvement at week 16; loss to follow-up at week 28.	NR	7 months
	PPP (palmoplantar) and PV	Case series (3/5)	Male, 51 years	Alitretinoin and adalimumab	Deucravacitinib 6 mg 1×/day and methotrexate 15 mg/week	Exacerbation in weeks 4 and 16; exacerbation of ankylosing spondylitis.	NR	4 months
	PPP (palmoplantar), PV and PsA	Case series (4/5)	Female, 41 years	Adalimumab and secukinumab	Deucravacitinib 6 mg 1×/day and methotrexate 15 mg/week	Exacerbation in week 4; improvement in week 16.	NR	12 months
	PPP (palmoplantar), PV and PsA	Case series (5/5)	Female, 31 years	Phototherapy, methotrexate, cyclosporine, bimekizumab, certolizumab, and guselkumab.	Deucravacitinib 6 mg/day and PUVA cream in week 12	Improvement in week 16; failure in week 48.	Palpitations, herpes simplex, cystitis, and folliculitis in three of five cases.	12 months
Choi et al., 2025^8^	NPPP (palmoplantar) and PV	Case series (1/2)	Female, 61 years	Topical drugs, phototherapy, acitretin, and secukinumab.	Upadacitinib 15 mg 1×/day	Resolution within 3 months	Elevated triglycerides (2.24 mmoL/L)	3 months
	NPPP (palmoplantar)	Case series (2/2)	Female, 52 years	Topical drugs, alitretinoin, apremilast, cyclosporine, ixekizumab, risankizumab, secukinumab, and ustekinumab.	Upadacitinib 15 mg 1×/day	Pruritus improvement within 2 weeks; resolution within 3 months.	Elevated ALT (124 U/L)	3 months
Hardy et al., 2025^9^	NPPP (palmoplantar) and AA associated with CVID	Case report	Female, 33 years	Topical drugs	Upadacitinib 15 mg 1x/day	Improvement within 2 weeks; resolution within 12 weeks.	None	20 months
Present case	NPPP	Case report	Female, 48 years	Topical drugs, acitretin, methotrexate, adalimumab, and secukinumab.	Tofacitinib 5 mg 2×/day	Pruritus improvement within 2 weeks; resolution within 4 months.	Headache	12 months

PP, Palmoplantar Psoriasis; NPPP, Non-pustular palmoplantar psoriasis; PPP, Pustular Palmoplantar Psoriasis; PV, Psoriasis Vulgaris; PsA, Psoriatic Arthritis; AA, Alopecia Areata; CVID, Common Variable Immunodeficiency; XR, Extended Release; ALT, Alanine Aminotransferase; NR, Not Reported.

Despite the clinical-histopathological correlation of the presented case, compatible with NPPP, it is important to recognize that some forms of psoriasis show an exuberant inflammatory phenotype and eczematous areas, which may simulate or even coexist with chronic palmoplantar eczema. The distinction between these entities is challenging, especially when there is clinical overlap of fissures, erythema, pruritus, and hyperkeratosis. In these contexts, the diagnostic spectrum should include both NPPP and hyperkeratotic eczema of the palms and soles, whose differentiation may require an exercise in clinical, histopathological, and therapeutic response correlation.^11^ Furthermore, there are reports of modification of the psoriasis vulgaris phenotype to eczematous conditions after therapies with biologics.^12^ Therefore, JAKi, by modulating multiple inflammatory pathways (such as IL-4, IL-13, IL-22, and IFN-γ), constitutes a therapeutic alternative considering the immunopathological complexity of these presentations.^13^

Regarding safety, tofacitinib requires vigilance regarding infections, updated vaccination status, and drug interactions. Caution should be exercised in patients with renal or hepatic failure, thrombophilia, cardiovascular risk, and neoplasms. Monitoring tests include complete blood count, lipid profile, liver and kidney function tests, and screening for viral and bacterial infections.^14^

Although oral tofacitinib has a satisfactory safety and efficacy profile, it has not been approved for psoriasis by the Food and Drug Administration due to side effects and the availability of other therapies. However, it was approved in 2012 for rheumatoid arthritis and subsequently for psoriatic arthritis, ulcerative colitis, juvenile idiopathic arthritis, and ankylosing spondylitis. Deucravacitinib is the only JAKi approved for moderate to severe plaque psoriasis; however, there are no comparative studies evaluating other JAKi (e.g., tofacitinib, upadacitinib, abrocitinib, baricitinib) in the treatment of NPPP.

This case documents a significant clinical response in refractory NPPP, corroborating the therapeutic potential of JAKi and reinforcing the need for controlled studies for this specific phenotype.

## ORCID IDs

Paula Hitomi Sakiyama: 0000-0001-7813-8294

Luciane Donida Bartoli Miot: 0000-0002-2388-7842

## Research data availability

Does not apply.

## Financial support

CNPq (306358/2022-0) – Hélio Amante Miot is a research fellow of CNPq.

## Authors' contributions

Paula Hitomi Sakiyama: Design and planning of the study; collection, analysis, and interpretation of data; drafting and editing of the manuscript; critical review of important intellectual content; critical review of the literature; approval of the final version of the manuscript.

Luciane Donida Bartoli Miot: Design and planning of the study; collection, analysis and interpretation of data; drafting and editing of the manuscript; critical review of important intellectual content; effective participation in research orientation; intellectual participation in the propaedeutic and/or therapeutic conduct of the studied cases; critical review of the literature; approval of the final version of the manuscript.

Hélio Amante Miot: Design and planning of the study; collection, analysis, and interpretation of data; drafting and editing of the manuscript; critical review of important intellectual content; effective participation in research orientation; intellectual participation in the propaedeutic and/or therapeutic conduct of the studied cases; critical review of the literature; approval of the final version of the manuscript.

## Conflicts of interest

None declared.
